# Self-monitoring Using Mobile Phones in the Early Stages of Adolescent Depression: Randomized Controlled Trial

**DOI:** 10.2196/jmir.1858

**Published:** 2012-06-25

**Authors:** Sylvia Deidre Kauer, Sophie Caroline Reid, Alexander Hew Dale Crooke, Angela Khor, Stephen John Charles Hearps, Anthony Francis Jorm, Lena Sanci, George Patton

**Affiliations:** ^1^Centre for Adolescent HealthRoyal Children's Hospital & Murdoch Childrens Research InstituteParkville, VICAustralia; ^2^School of Behavioural ScienceUniversity of MelbourneMelbourne, VICAustralia; ^3^Orygen Youth Health Research CentreCentre for Youth Mental HealthUniversity of MelbourneParkville, VICAustralia; ^4^Department of General PracticeUniversity of MelbourneCarlton, VICAustralia; ^5^Department of PaediatricsUniversity of MelbourneMelbourneAustralia

**Keywords:** Mobile phone, early intervention, patient monitoring, randomized controlled trial, consciousness, depressive disorder, affect

## Abstract

**Background:**

The stepped-care approach, where people with early symptoms of depression are stepped up from low-intensity interventions to higher-level interventions as needed, has the potential to assist many people with mild depressive symptoms. Self-monitoring techniques assist people to understand their mental health symptoms by increasing their emotional self-awareness (ESA) and can be easily distributed on mobile phones at low cost. Increasing ESA is an important first step in psychotherapy and has the potential to intervene before mild depressive symptoms progress to major depressive disorder. In this secondary analysis we examined a mobile phone self-monitoring tool used by young people experiencing mild or more depressive symptoms to investigate the relationships between self-monitoring, ESA, and depression.

**Objectives:**

We tested two main hypotheses: (1) people who monitored their mood, stress, and coping strategies would have increased ESA from pretest to 6-week follow-up compared with an attention comparison group, and (2) an increase in ESA would predict a decrease in depressive symptoms.

**Methods:**

We recruited patients aged 14 to 24 years from rural and metropolitan general practices. Eligible participants were identified as having mild or more mental health concerns by their general practitioner. Participants were randomly assigned to either the intervention group (where mood, stress, and daily activities were monitored) or the attention comparison group (where only daily activities were monitored), and both groups self-monitored for 2 to 4 weeks. Randomization was carried out electronically via random seed generation, by an in-house computer programmer; therefore, general practitioners, participants, and researchers were blinded to group allocation at randomization. Participants completed pretest, posttest, and 6-week follow-up measures of the Depression Anxiety Stress Scale and the ESA Scale. We estimated a parallel process latent growth curve model (LGCM) using Mplus to test the indirect effect of the intervention on depressive symptoms via the mediator ESA, and calculated 95% bias-corrected bootstrapping confidence intervals (CIs).

**Results:**

Of the 163 participants assessed for eligibility, 118 were randomly assigned and 114 were included in analyses (68 in the intervention group and 46 in the comparison group). A parallel process LGCM estimated the indirect effect of the intervention on depressive symptoms via ESA and was shown to be statistically significant based on the 95% bias-corrected bootstrapping CIs not containing zero (–6.366 to –0.029). The proportion of the maximum possible indirect effect estimated was κ^2 ^=.54 (95% CI .426–.640).

**Conclusions:**

This study supported the hypothesis that self-monitoring increases ESA, which in turn decreases depressive symptoms for young people with mild or more depressive symptoms. Mobile phone self-monitoring programs are ideally suited to first-step intervention programs for depression in the stepped-care approach, particularly when ESA is targeted as a mediating factor.

**Trial Registration:**

ClinicalTrials.gov NCT00794222; http://clinicaltrials.gov/ct2/show/NCT00794222 (Archived by WebCite at http://www.webcitation.org/65lldW34k)

## Introduction

Depression is a common, recurrent disorder and contributes to a substantial burden of disease [1]. Up to 25% of the population experience at least one episode of depression in their lifetime [2] with more women affected by depression than men [2,3]. First episodes of depression generally begin during adolescence [4] with up to 30% of young people experiencing mild subclinical depressive symptoms by 18 years of age [5,6] and many progressing to major depressive disorder [6]. A meta-analysis of the depressive disorder point prevalence estimates for 13- to 18-year-olds reported that 5.7% of adolescents may have a depressive disorder diagnosed at any one point in time [7]. The severity and longevity of depression are associated with substantial economic costs [8], attributed to the early onset and recurrent nature of depression. The World Health Organization predicted that depression will be the second leading cause of disease burden worldwide by 2020 [1], indicating the need for new methods to reduce the burden of depression. Current interventions are estimated to cover 66% of the burden of depression, with 33% of this burden unable to be averted by available treatments [4]. The large number of people who experience mild depressive symptoms account for more burden of depression than those with major depressive disorder [9], suggesting that new methods are needed and that they should focus on the early stages of depression.

The stepped-care approach, beginning with simple, inexpensive interventions (that are given before the onset of diagnosed major depressive disorder) with the ability to step up to higher-intensity programs as needed [9-11], has the advantage of providing a low-intensity early intervention with reduced length and cost of the treatment to young people experiencing mild mental health symptoms [12,13]. Early interventions that focus on intensive computerized or school-based cognitive behavioral therapy-based programs may be best regarded as second-step interventions, as they are time consuming and have high attrition rates [14]. Third-step interventions may include individualized face-to-face therapy, whereas a combination of face-to-face therapy and antidepressants should be considered only as a last resort for teenagers [11]. Van Straten et al [11] advocate the use of watchful waiting as a first step in the stepped-care model. The watchful waiting approach has two limitations. First, as general practitioners (GPs) are often the first point of contact for people with mild mental health symptoms, the onus of watchful waiting would be placed on GPs. GPs, however, are under pressure to treat many people within a day and to keep appointment times brief (the current average appointment time is 15 minutes) [15]. Second, a young person’s lack of awareness of emotional distress may reduce the effectiveness of the watchful waiting approach, as GPs are more likely to identify mental health problems in young people who are aware of emotion distress [16]. Short programs involving the completion of homework diaries have been shown to have larger effects on depressive symptoms than longer-duration programs do and offer a potential alternative first-step approach to watchful waiting [17]. Such programs may be completed via mobile phones as a first-step early intervention with the opportunity to step up to more intensive treatment programs as needed.

### Mobile Phone Self-monitoring Program

Methods for stepped-care interventions involving technology, such as computers, the Internet, or mobile phones, and simple methods such as self-monitoring techniques can engage young people and foster their involvement [18-20]. Technology is particularly important when targeting young people, dubbed early adopters, as they are familiar with and rely on technology for much of their social interactions with peers and readily engage with electronic devices [18-20]. In particular, studies conducted using electronic self-monitoring devices with young people have demonstrated that young people readily engage with the technology [21-23] and have high compliance [24].

We developed and piloted a mobile phone self-monitoring program, the mobiletype program (Mobile Tracking of Young People’s Experiences) [21,23,25], well suited as a first step in the stepped-care approach. In our pilot studies, young people were asked to monitor their mood, stress, and daily activities on a mobile phone application 4 times a day for 1 week. Results suggested that young people complied with self-monitoring of mental health symptoms for the purpose of reviewing these data with their GP and found it a simple, easy-to-use tool to track their daily experiences. Both GPs and patients found communicating information via the mobiletype program to be beneficial, and the program enabled GPs to better understand the young person’s mental health. The self-monitoring data can be uploaded to GPs and used to detect depressive symptoms and other mental health information to allow progression to further higher-intensity intervention if needed. Self-monitoring approaches are typically used in psychotherapy as an adjunct to cognitive behavioral therapy, with research demonstrating that self-monitoring increases the benefits of therapy more than therapy alone [26]. Research focusing on momentary sampling techniques demonstrates that self-monitoring itself can lead to a change in behavior and that changes are generally made in a favorable direction [27-29].

### Mechanism Underlying Self-monitoring

The contents of early intervention programs generally target a mechanism that predicts the outcome [30]. For example, cognitive–behavioral-based universal programs focus on cognitive restructuring and problem-solving skills training [31], which are predicted to reduce depressive symptoms. In this study, the intervention was based on emotional self-awareness (ESA), which is hypothesized to predict depressive symptomatology. Increasing awareness of emotions is an important therapeutic step in most psychotherapies for depression and other mental illnesses [32] by preparing individuals for changing their cognitions, beliefs, and schemas [33]. A recent case study using mobile phones [34] showed that self-monitoring increased positive mood and coping strategies while decreasing negative mood in four adult employees with stress. Participants’ ESA increased, and they were able to internalize the questions and therapies used in the mobile program on completion of the study. Self-monitoring studies are promising; however, further research is needed to explore the possible mental health benefits of self-monitoring techniques with young people who have symptoms of depression.

ESA may also provide a suitable framework for first-step intervention programs by assisting young people to become aware of their emotions in preparation for learning more adaptive coping strategies. Adolescence is an ideal target for first-step intervention of mental health problems, as young people begin to develop the ability to independently cope with everyday stresses and negative emotions during their teen years [35]. The current study focused on examining the relationship between self-monitoring and depressive symptoms through the mechanism of ESA, specifically focusing on (1) recognizing emotions [32,36,37], (2) being able to identify emotions [38-40], (3) identifying contextual factors surrounding emotions [36,38,41], (4) communicating emotional states and associated factors to others and internally [42-44], and (5) planning and making decisions about how to cope with an emotional state [38,45,46]. Through self-monitoring, young people can learn to recognize emotional states, and to identify and differentiate various emotions within different contexts, leading to effective communication of emotions to others and productive decision making.

The overall aim of the current study was to investigate, in a randomized controlled trial, the utility of the mobiletype program as a first-step intervention program. The primary hypothesis was that young people who completed the mobiletype intervention program would have lower depressive symptoms than those who completed the attention comparison program. Using a mixed-methods model, we found that depressive symptoms significantly decreased over time for both intervention and attention comparison groups, and we found no significant difference in mental health symptoms between groups [47]. We attributed the lack of difference between groups in mental health symptoms to the unanticipated effect of the training, resources, and support given to young people and GPs over the course of the study for both the intervention and the comparison groups. Interestingly, the intervention group was found to have a significant increase in ESA compared with the comparison group. As both groups were found to have significantly decreased mental health symptoms, there was reduced power to detect a difference between groups.

The goal of this secondary analysis was to further examine the effects of a mobile phone self-monitoring program on ESA among young people with mild or more depressive symptoms as a first-step treatment of depression. Specifically, we were interested in the effects of self-monitoring on ESA and the association between ESA and depressive symptoms. We hypothesized that self-monitoring mood, stress, and coping strategies would increase young people’s awareness of their emotions, which would lead to a decrease in their symptoms of depression. We tested the following hypotheses: (1) young people in the intervention group would have an increase in ESA from pretest to 6-week follow-up compared with the attention comparison group, and (2) the increase in ESA would predict a decrease in depressive symptoms. We estimated a parallel process latent growth curve model (LGCM) [30,48] to investigate whether ESA would mediate the relationship between the intervention program and depressive symptoms. A secondary analysis compared the effects of the mobiletype intervention and comparison programs on rumination, with the hypothesis that the intervention group would have decreased rumination compared with the comparison group.

## Methods

### Trial Design

The data presented here were from the mobiletype randomized controlled trial conducted from 2009 to 2011. This was a multicenter, multiregional, stratified (according to region), single-blind, attention-controlled study with balanced (1:1) individual randomization into parallel groups. This study was conducted in Victoria, Australia adhering to the reporting recommendations from the CONSORT statement [49] as a guide.

### Participants

#### General Practitioners

All GPs in the Goulburn Valley Region and Albury-Wodonga Region were invited to participate in the study via the Regional Division of General Practice (support units that service clinical practices within a region). GPs in Melbourne were recruited via the local Divisions of General Practice. Clinics were targeted that listed an interest in adolescent health on the Melbourne General Practice Network (www.mgpn.com.au). Participating GPs were trained to use the mobiletype website and were provided with a study manual that included the study procedure, a variety of clinical supports (including referral details of adolescent-friendly allied health professionals and services), youth-friendly Internet, email, and phone support, and youth-focused psychoeducation handouts on a range of mental health problems (this information was also available on the mobiletype website), which were available for all participating GPs and patients. Continuing professional development quality assurance points were available to GPs for their participation in the study. Of the 103 GPs who agreed to participate, 35 actively participated in the study with at least one young person. These contributing GPs were from 26 different practices in the three recruitment areas: 12 in greater Melbourne, 7 in Albury-Wodonga, and 7 in the Goulburn Valley.

#### Young People

Young people meeting the following inclusion criteria were eligible to participate regardless of their reason for visiting the GPs. Participants were required to (1) be aged between 14 and 24 years, (2) speak proficient English, and (3) have a mild or more severe emotional or mental health issue as assessed by their GP or indicated by a score greater than 16 on the Kessler Psychological Distress Scale [50]. Participants were excluded if their psychiatric or medical condition prevented them from complying with either the requirements of informed consent or the study protocol (ie, current psychosis or imminent hospitalization).

### Mobiletype Program

We used Version 4 of the mobiletype program as the intervention and attention comparison in this study, which was created in-house using Java Platform, Micro Edition by the Murdoch Childrens Research Institute. This program was written for use with multiple models of mobile phones and firmware. For this trial, participants were lent a study mobile phone with either the mobiletype intervention or a comparison program uploaded onto it. Data from the program was uploaded to a secure website constructed and hosted by the Murdoch Childrens Research Institute as well as encrypted and stored on the mobile phones.

Participants were prompted to complete a mobiletype entry by an auditory signal (beep) emitted from the mobile phone at random intervals in the blocks outlined in Table 1. If no report was completed the phone emitted one reminder signal after 5 minutes. Entries were time coded and saved. Participants were also able to complete the program any time and were able to complete an entry between 10 PM and 8 AM, although no trigger was sent at this time. Entries from 10 PM to 12 AM consisted of the evening questions and entries from 12 to 8 AM consisted of the same questions as the afternoon questions, as shown in Table 1. Each report took approximately 1–3 minutes to complete.

#### Intervention Program

The intervention group monitored themselves using the complete mobiletype program, which assessed 8 areas of functioning as developed in previous mobiletype studies [21,23], consisting of current activities, location, companions, mood, recent stressful events, responses to stressful events, alcohol use, cannabis use, quality and quantity of sleep, quantity and type of exercise, and diet. The time of day when each module assessing the eight areas was delivered varied as displayed in Table 1.

**Table 1:** Modules included in each block of the mobiletype comparison and intervention programs.

Module		Morning 8–10:59 AM	Noon 11 AM to 3:29 PM	Afternoon 3:30–7:59 PM	Evening 8–10 PM
**Intervention program**					
	Current activity	✓	✓	✓	✓
	Stress	✓	✓	✓	✓
	Mood	✓	✓	✓	✓
	Alcohol use		✓		
	Cannabis use		✓		
	Sleep	✓			
	Diet				✓
	Exercise				✓
**Comparison program**					
	Current activity	✓	✓	✓	✓
	Stress				
	Mood				
	Alcohol use				
	Cannabis use				
	Sleep	✓			
	Diet				✓
	Exercise				✓

#### Comparison Program

The attention comparison program was designed to provide a data collection process similar to that in the intervention group by controlling for the amount of time spent engaged in the program condition and the overall research methodology and the attention given to them by health care professionals and research staff [51]. The comparison group monitored themselves using an abbreviated version of the mobiletype program, which assessed only current activities, location, companions, quality and quantity of sleep, quantity and type of exercise, and diet (meals, snacks, junk food, and soft drinks consumed). Importantly, we removed the modules pertaining to ESA and mental health as shown in Table 1 (ie, mood, stress, alcohol and cannabis use).

#### Summary Reports

Data collected by the mobiletype program (intervention and comparison groups) on the mobile phone was sent via short message service to a secure website, where it was automatically collated. Each area of assessment was displayed in graphs (eg, daily mood graphs) or in tables (eg, daily alcohol intake). An individualized summary report of the data was written following structured prescriptive guidelines by the second author (registered psychologist), or the first author under the supervision of the second author.

### Outcome Measures

The pretest, posttest, and 6-week follow-up questionnaire packages included the Depression Anxiety Stress Scale (DASS) [52] and the ESA Scale. The DASS is a 21-item response form with subscales of depression, anxiety, and stress with scores ranging from 0 to 42. The DASS has Australian norms and clinically validated ranges. A high DASS score indicates greater levels of depression, anxiety, or stress symptoms. The ESA Scale was adapted from the 20-item Self-reflection and Insight Scale [53], the 10-item Ruminative Response Scale [54], and the 12-item Meta-Evaluation Scale [46]. As there is no overall measure of ESA that covers the five areas of recognition, identification, communication, contextualization, and decision making, we adapted 33 items pertaining to these areas from the above scales (see Multimedia Appendix 1). These were then combined to create a total ESA scale with high internal consistency (Cronbach alpha = .83). The total ESA score ranged from 0 to 132, with higher scores indicating more ESA. Rumination was measured by the brooding subscale of the Ruminative Response Scale, which consisted of 5 items ranging from 0 to 4. A higher score indicates higher rumination.

### Sample Size

We anticipated recruitment of 200 participants from 10 general practices. This sample size was based on Cohen’s [55] statistical testing for multiple regression with two independent variables (accounting for the mediating variable and the outcome) to detect a medium effect with 80% power and a probability of a type I error of .05. We selected a medium effect size, as we considered this to be clinically significant. Using Fritz and MacKinnon’s statistical tests [56], a sample size of 71 participants inclusive should be sufficient to detect a medium mediation effect with 80% power with bias-corrected bootstrapping (see below). The anticipated sample size of 200 was not met due to delays in recruitment during school holidays and the H1N1 influenza pandemic [57]. As a result, we set a deadline for stopping recruitment and recruited a total of 118 participants.

### Randomization

Participants were randomly assigned to either (1) the mobiletype monitoring intervention group or (2) the mobiletype attention comparison group; both groups also received medical care as usual. A database was set up by an in-house computer programmer with identification numbers for 100 Melbourne, 50 Goulburn Valley, and 50 Albury-Wodonga participants. Each number was attached to a link that downloaded either the intervention or comparison program directly to the mobile phone. This process was blinded; the intervention and comparison program could not be differentiated when downloading the program. The programmer used a random seed generator to allocate each program to the 200 identification numbers at the individual level and stratified according to area (Melbourne, Goulburn Valley, and Albury-Wodonga). This programmer was not involved in any data collection or analysis. A research assistant downloaded each program by selecting the next consecutive link for the next study mobile phone and was blinded to the allocation, as he knew only the identification number and area to load onto study mobile phones (eg, Melbourne01, Melbourne02). Mobile phones and identification numbers were allocated to consecutively recruited participants. The researchers, participants, and GPs were blinded to randomization pretest. GPs and participants became aware of the group allocation at the posttest when the summary reports were reviewed. This study had approval from the Human Research Ethics Committee of the Royal Children’s Hospital, Melbourne (RCH HREC: 28113), and was registered in ClinicalTrials.gov (Reference: NCT00794222).

### Procedure

#### Recruitment

In addition to providing treatment as usual, GPs screened their patients for eligibility to the study; organized an appointment for willing participants with a research assistant using an online booking form or a faxed referral form, or by phone; and completed a pretest questionnaire for each participant. Participants then met with a mobiletype research assistant, generally within 5 days of referral, to learn the study process, complete consent forms and the pretest questionnaire package, familiarize themselves with the mobiletype program and the other features of the phone, and complete a practice entry of the mobiletype program. Participants were provided with a study manual that described the research procedure and offered trouble-shooting tips.

#### Mobile Phone Monitoring Period

All participants borrowed a Sony Ericsson 7501i (Sony Limited Australia, North Ryde, NSW, Australia) mobile phone containing the mobiletype program for the study period. Information regarding the development and testing of the mobiletype program has been previously published [21]. Participants were requested to complete at least two mobiletype entries a day until they returned for their medical review in 2 to 4 weeks. Participants and GPs were advised that 2 to 4 weeks’ monitoring was the ideal monitoring period. Participants were given a subscriber identity module (SIM) card containing A$30 in credit as partial reimbursement for their time and phone credit used.

#### Posttest Review

On completing the monitoring period, participants reviewed the self-monitoring data with their GP on the mobiletype website. Young people completed a posttest assessment immediately following this appointment, and again at 6 weeks and 6 months after this review (6-month posttests not included in the current analysis). GPs completed a posttest questionnaire immediately after the appointment. Questionnaires were completed online, over the phone with a researcher, or via a mailed hardcopy questionnaire. Participants were given a A$20 gift card for each posttest questionnaire completed (maximum of A$60 for all questionnaires completed).

### Analyses

We conducted all analyses on an intention-to-treat basis using all available data from participants included at randomization. Data were assumed to be missing at random [58], and maximum likelihood estimation was used with missing data accounted for by the missing routines in the statistical program used.

#### Parallel Process Latent Growth Curve Model

Recently, structural equation modeling has modernized Baron and Kenny’s well-known mediation model [59,60]. LGCM is a structural equation modeling technique that allows for examination of inter- and intrapersonal changes and, importantly, parallel process LGCM accommodates longitudinal data for situations in which both the mediator and outcome change over time [61]. Baron and Kenny’s requirement of a statistically significant pathway between the independent variable and the outcome [59] is no longer recommended, as detecting a total effect between the independent variable and outcome reduces power, therefore reducing the likelihood that a mediated effect can be detected [56,62]. Parallel process LGCM based on Cheong et al [30] was used to test the hypothesis that the mobiletype program would increase ESA and that the change in ESA over time would change (decrease) depressive symptoms, by using the software program Mplus Version 6.11 (Muthén & Muthén, Los Angeles, CA, USA). The model estimates two latent variables, the intercept (estimated starting point) and the slope (estimated growth curve), using repeated measures for the mediator (ESA) and the outcome (depression) across the three time points. The latent intercept for both ESA and depression had factor loadings set to 1 to represent the starting point of the growth trajectory, and the factor loadings of the latent linear slope were fixed to 0, 3, and 9 to represent the time between tests: pretest (week 0), posttest (week 2–4), and 6-week follow-up (week 8–10), respectively. Group was defined as 0 for the comparison group and 1 for the intervention group. A combination of at least three of the following cut-off values was used to determine goodness of fit of the model as recommended [63,64]: comparative fit index > .95, Tucker-Lewis Index > .95, standardized root mean square residual < .08, and root mean square error of approximation and root mean square error of approximation < .05. Another statistic considered was chi-square divided by degrees of freedom, with values less than 2 indicating a good fit and values up to 3 indicating an acceptable fit [64]. When specifying the model, we allowed the variances of the observed variables to covary at each time point and calculated all regression pathways. Indirect effects of group on the slope of depression via the intercept and the slope of ESA were also specified. As recommended [56,62,65], we used bootstrapping with resampling at 5000, with bias-corrected confidence intervals reported for the indirect pathway [66]. Resampling provides more accurate results with fewer assumptions about the data (such as normal distributions) [60].

#### Indirect Effect Sizes

A recent development in statistical analysis has led to the capacity of estimating effect sizes for mediation models [67]. Using R for Mac OS X GUI 1.40-devel, an open-source language and environment for statistical analysis (http://www.r-project.org/), we calculated effect sizes of the indirect pathways using the mediation function in the MBESS (http://cran.r-project.org/web/packages/mediation/mediation.pdf) in R. Bootstrapping was used to calculate 95% confidence intervals. As recommended [60,67], both the unstandardized indirect effect size and the proportion of the maximum possible indirect effect (κ^2^) are reported and described in the results. Other estimates of effect size obtained in this analysis are listed in Multimedia Appendix 2.

#### Rumination

We conducted a secondary analysis to determine whether the intervention group had a decrease in rumination compared with the comparison group. A mixed model analysis was conducted over time and between groups using SPSS Version 17.0.0 (IBM Corporation, Somers, NY, USA) with the MIXED procedure. As with the LGCM, survey time was entered as a continuous variable in weeks (0, 3, and 9). The mixed model employed the more conservative restricted maximum likelihood estimation and unstructured covariance matrix.

## Results

### Recruitment

**Figure 1:**
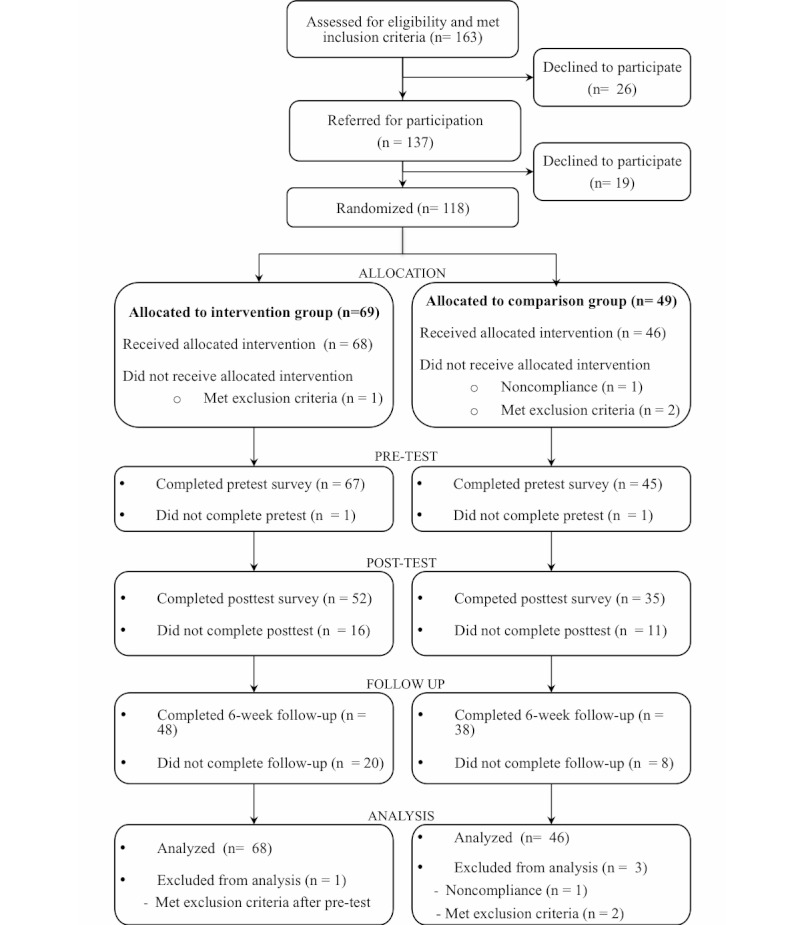
Flow diagram of the study process.

We collected data collection between April 16, 2009 and January 28, 2011. As seen in Figure 1, 137 young people accepted the invitation to join the study, of whom 118 began the recruitment process. We excluded 4 participants after randomization (2 became too unwell to participate, 1 was incarcerated, and 1 gave invalid responses to all pretest measures), resulting in a final sample of 114 young people, which was sufficient to detect the primary aim of a medium-sized indirect effect [56]. Due to a failure to recruit the expected sample of 200 participants, there is a different number of participants in the comparison and intervention groups; however, a test of the binomial distribution indicated that this difference was not significant with 69 of 118 participants randomly allocated to the intervention group (*P *= .08). The total number of participants assessed for eligibility was difficult to establish, as the GPs rarely recorded information about patients who met the inclusion criteria and were either not approached to participate or declined when invited to participate. Therefore, the number of patients assessed for eligibility presented in Figure 1 is likely to be underrepresented.

In total, 67% of participants (76/114) completed all questionnaires and 85% of participants (97/114) completed questionnaires at two or more time points. We conducted *t *tests and chi-square tests and found no significant differences between participants who completed all questionnaires and those who missed questionnaires. Therefore, we included all 114 participants (68 in the intervention group and 46 in the comparison group) in analyses using the routines for missing data in the maximum likelihood estimation. Of 114 participants, 2 (2%) did not complete the pretest questionnaire but went on to complete the mobiletype entries and posttests. We could not contact 27 participants (24%) for the posttest questionnaire but they went on to complete the 6-week follow-up questionnaire.

### Demographics

We found no statistically significant differences in demographic characteristics between the intervention and comparison groups on any pretest measures listed in Table 2, except that the intervention group reported significantly higher stress than the comparison group with a mean difference of 3.4 (*t*_109 _= 2.06, *P *= .04).

Participants in the intervention group completed an average of 3.3 (SD 1.42, range 1–8) mobiletype entries each day and completed the program from 1 to 34 days with a mean of 17.7 (SD 6.69) days completed. In the comparison group, participants completed an average of 4 (SD 1.77, range 1–12) mobiletype entries per day, and completed the program for 8 to 25 days with a mean of 16.8 (SD 4.03) days completed. The minimum dose of the program was completion of at least two mobiletype entries a day for at least 14 days. As Table 2 shows, 64 (56%) participants in intervention group and 36 (52.9%) in comparison received a minimum dose. Means, standard deviations and 95% confidence intervals are presented in Table 3 using the observed scores.

**Table 2:** General demographics of participants in the comparison and intervention groups (n = 114).

Characteristic		Comparison group	Intervention group	*P *value
Total number^a^, n (%)		49 (41.5%)	69 (58.5.0%)	.08
14 days completed^b^, n (%)		28 (59.6%)	36 (52.9%)	.33
**Area**, n (%)				
	Melbourne	14 (30.4%)	28 (42.6%)	.27
	Goulburn Valley	21 (45.7%)	21 (29.4%)	
	Albury-Wodonga	11 (23.9%)	19 (27.9%)	
Male participants, n (%)		17 (37.0%)	15 (22.1%)	.08
Age (years), mean (SD)		17.4 (3.2)	18.5 (3.2)	.06
Ethnic identification^c^, n (%)		4 (9.1%)	10 (22.7%)	.37
**Employment**, n (%)				
	Employed	7 (15.2%)	18 (26.5%)	.21
	Unemployed	4 (8.7%)	9 (13.2%)	
	Student	35 (76.1%)	41 (60.3%)	
**Drug-related items** ^c^				
	Ever had alcohol	38 (86.4%)	59 (88.1%)	.79
	Ever been drunk	31 (70.5%)	53 (79.1%)	.30
	Ever had a cigarette	25 (56.8%)	38 (56.7%)	.99
	Ever tried marijuana	18 (40.9%)	33 (49.3%)	.39
	Ever tried other^d ^drugs	10 (22.7%)	26 (38.8%)	.08
**Pretest DASS** ^c,e^, mean (SD)				
	Depression	19.4 (10.8)	20.4 (11.0)	.63
	Anxiety	11.0 (8.0)	14.1 (9.7)	.08
	Stress	16.9 (7.9)	20.3 (8.9)	.04

^a ^Binomial test on number at randomization (n = 118).

^b ^Completed mobiletype entries at least twice daily for 14 days.

^c ^Observed means (n = 111).

^d ^Sedatives, tranquilizers, amphetamines, analgesics, inhalants, cocaine, LSD, and heroin.

^e ^Depression Anxiety Stress Scale.

**Table 3:** Descriptive statistics for the intervention and comparison groups’ scores^a ^on depression and emotional self-awareness at pretest, posttest, and 6-week follow-up.

		Comparison group			Intervention group		
		n^b^	Mean (SD)	95% CI	n^b^	Mean (SD)	95% CI
Depression							
	Pretest	44	19.4 (10.9)	16.1–22.7	67	20.4 (11.0)	17.8–23.1
	Posttest	33	15.2 (8.9)	12.1–18.3	50	16.3 (10.8)	13.3–19.4
	6-week follow-up	36	12.5 (11.8)	8.5–16.5	50	13.5 (10.5)	10.5–16.5
Emotional self-awareness							
	Pretest	44	61.1 (11.9)	57.4–64.7	67	61.6 (12.1)	58.7–64.6
	Posttest	32	63.1 (11.1)	59.1–67.1	46	64.7 (10.9)	60.9–67.4
	6-week follow-up	35	62.2 (11.6)	58.2–66.1	47	68.9 (11.2)	65.5–72.1
Rumination							
	Pretest	44	12.8 (3.16)	11.9–13.8	67	14.0 (3.43)	13.2–14.9
	Posttest	33	12.2 (3.57)	10.9–13.4	46	12.4 (3.57)	11.3–13.4
	6-week follow-up	35	11.2 (3.67)	10.0–12.5	48	11.7 (3.62)	10.7–12.8

^a ^Observed scores.

^b ^Number of participants used to calculate the mean, standard deviation (SD), and 95% confidence interval (CI).

### Parallel Process Latent Growth Curve Model

**Figure 2:**
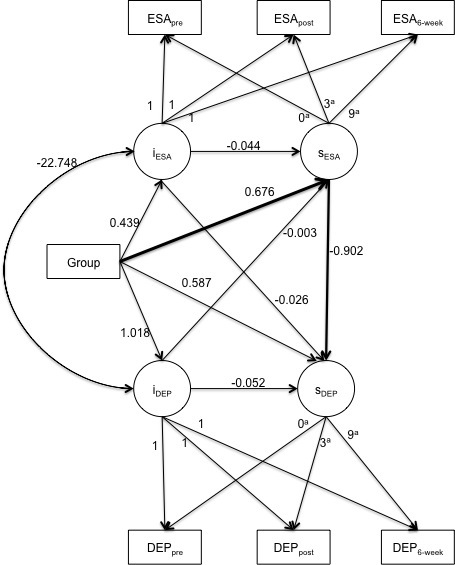
Parallel process latent growth curve model of the group effect on the growth of depressive symptoms via the growth of emotional self-awareness (ESA). Unstandardized estimates reported; boldface lines represent statistically significant pathways. ^a^Time interval from pretest by week. DEP_pre, post, 6-week_, ESA_pre, post,6-week_ = the observed score of the Depression Anxiety Stress Scale depression subscale and ESA scale at pretest, posttest, and 6-week follow-up, respectively; Group = intervention program condition; iDEP = latent intercept of depression; iESA = latent intercept of ESA; sDEP = latent slope of depression; sESA = latent slope of ESA.

The path diagram in Figure 2 shows the parallel process LGCM used to test the indirect pathway of the mobiletype program used on depressive symptoms via ESA. The fit indices for Figure 2 suggest that the model is a good fit for the data (χ^2^_6 _= 11.3, *P *= .08, comparative fit index = .958, Tucker-Lewis Index = .854, root mean square error of approximation = .088, *P *= .18, standardized root mean square residual = .040).

All possible pathways were calculated between group and the four latent variables as illustrated in Figure 2. The first step to testing mediation was conducted by regressing the slope of ESA onto group. The positive coefficient indicates that there was a greater increase in ESA across time in the intervention group than in the comparison group. This coefficient was statistically significant, as the 95% bias-corrected bootstrap CIs of group on ESA did not contain zero (see Table 4). The second step to testing mediation was conducted by regressing the slope of ESA onto the slope of depression. The negative coefficient here indicates that an increase in ESA led to a decrease in depressive symptoms. This pathway was also statistically significant as indicated by the 95% bias-corrected bootstrap CIs not containing zero (see Table 4). All other pathways illustrated in Figure 2 were tested and were not statistically significant.

The indirect effect of group on the slope of depression via the slope of ESA reported in Table 4 is statistically significant (95% bootstrap CI did not contain zero) with an unstandardized estimate of –0.608, indicating that participants in the intervention group had a decrease in depressive symptoms via the slope of ESA when compared with the comparison group. There was no significant direct effect from group to depressive symptoms based on the 95% bootstrap CI, indicating that the intervention did not directly decrease depressive symptoms.

**Figure 3:**
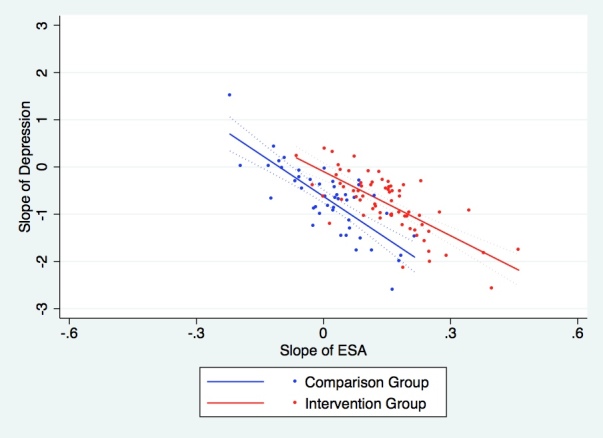
The relationship between self-monitoring, the slope of depressive symptoms, and the slope of emotional self-awareness (ESA). The points represent individuals' estimated slopes and the lines represent the line of best fit with 95% confidence intervals.

Figure 3 presents the relationship between the change in depressive symptoms and the change in ESA for both the intervention and comparison groups.

There was a negative relationship between changes in ESA and changes in depressive symptoms for both groups; accordingly, an increase in ESA was associated with a decrease in depressive symptoms as seen in Figure 3. The intervention group had larger increases in ESA than did the comparison group, which was associated with a greater decrease in depressive symptoms.

**Table 4:** Coefficients and bias-corrected bootstrapping confidence intervals (CIs) of the parallel process latent growth curve model.

Effect		Point estimate	95% Bootstrap CI
**Direct effect on the slope of depression**			
	Slope of ESA	–0.902^a^	–6.209 to –0.052
	Intercept of depression	–0.052	–0.126 to 0.012
	Intercept of ESA	–0.026	–0.315 to 0.027
	Group	0.587	–0.114 to 5.072
**Direct effect on the slope of ESA**			
	Intercept of ESA	–0.044	–0.083 to 0.162
	Intercept of depression	–0.003	–0.038 to 0.063
	Group	0.676^a^	0.019 to 1.231
**Direct effect of group on intercept**			
	ESA	0.439	–3.904 to 4.562
	Depression	1.018	–2.980 to 5.208


**Indirect effect of group on slope of depression**			
	Via the slope of ESA	–0.610^a^	–5.596 to –0.003
	Via the intercept of ESA	–0.012	–0.526 to 0.105
	Total indirect effect	–0.621^b^	–6.269 to –0.036

^a ^Confidence interval does not contain zero.

### Indirect Effect Sizes

The unstandardized indirect effect size can be interpreted on the DASS depression subscale (on a scale from 0 to 42), indicating that the intervention group is estimated to have a linear decrease in depressive symptoms of .688 per week (95% CI –.962 to –.487) indirectly through mediation of the linear slope of ESA when compared with the comparison group. The proportion of the maximum possible indirect effect has similar properties to, and can be interpreted on a similar scale to, Cohen's *r* *2 *[67]; therefore, the estimated κ^2 ^of .54 (95% CI .426–.640) indicates a large size of indirect effect.

### Rumination

We conducted a secondary analysis to determine whether rumination decreased over time between groups. The mixed model analysis of the Ruminative Response Scale brooding subscale showed a significant main effect of time (β = –0.16, *P *=.02), indicating an overall decrease in rumination over time for both groups. There was no significant main difference between groups (β = 1.01, *P *=.11), nor was the interaction between group and time significant (β = –0.07, *P *=.39).

## Discussion

The current study examined the use of a mobile phone self-monitoring program on ESA with young people who had mild or more depressive symptoms, and supported the hypothesis that self-monitoring mood, stress, and coping strategies increases awareness of emotions. The second hypothesis that an increase in ESA would predict a decrease in depressive symptoms was also supported. Based on Preacher and Kelley’s proportion of the maximum possible indirect effect [67], there was a large effect of the intervention program on depressive symptoms indirectly via ESA.

This study supports previous research suggesting that simple self-monitoring techniques effectively increase self-awareness, in this case, awareness of one’s own emotions [34,68]. Metacognitions, such as self-awareness, are developed during early adolescence [35], and interventions can be developed that target young people’s ability to recognize emotions, identify emotional states, understand the contextualization of emotions, communicate this emotional knowledge, and plan and make constructive decisions about emotions. Increasing ESA is a core process in the early stages of therapy [32]. The current study demonstrates the potential for targeting ESA in first-step intervention strategies for young people with mild or more depressive symptoms. This randomized controlled trial was conducted with a view of representing a wide variety of young people who visit GPs with a range of medical and psychological problems and severity of problems. Therefore, the results of this study are applicable to this age group in general.

Self-monitoring techniques may provide an alternative to watchful waiting as a first-step intervention in the stepped-care approach. Mobile phones are ideally suited to this purpose, as the mobiletype program can be downloaded to patients’ own mobile phones to help young people understand and manage mild depressive symptoms. Detailed information about patients’ mental health in recent weeks is then uploaded to GPs in an easy-to-read format, saving time in appointments and allowing progression to more intensive second-step interventions when needed. Young people often do not recognize mental health problems [69] and instead attend GP clinics for somatic complaints rather than mental health symptoms [70]. Using self-monitoring techniques with young people who present with underlying somatic complaints may increase their ESA and help young people initiate treatment for depression.

Our secondary hypothesis that the intervention program participants would have a decrease in rumination when compared with those in the comparison program was not supported. Further research is needed to determine whether there is an inverse relationship between rumination and ESA. Nevertheless, rumination decreased over time as did depressive symptoms [47], further supporting the positive relationship between depression and rumination.

Primary care is a particularly difficult setting in which to conduct randomized controlled studies [71,72]. In this study, recruitment was delayed and ceased before the anticipated sample size was recruited due to the H1N1 influenza pandemic, first detected in Melbourne on May 9, 2009 [57], and during school holidays. These delays resulted in uneven numbers in the two groups. We strongly recommend allocating extra time for recruitment in primary care compared with other settings, particularly in youth-focused studies. The intervention program had no direct effect on depressive symptoms. One interpretation of these results is that there was reduced power for a direct effect given that depressive symptoms decreased significantly over time in both groups. It is possible that both groups had a decrease in depressive symptoms due to the resources, training, and support given to the GPs; however, a larger sample size, or a wait-list control group, would be needed to determine whether depressive symptoms differed between the groups [56,62]. Finally, Reid et al [47] detail other limitations: a cluster randomized controlled trial, in which GPs rather than patients are randomly allocated, may have been more appropriate but was rejected during the study design due to the difficulty in blinding GPs and participants to the randomization procedure; and participant heterogeneity in illness type, severity, and familiarity with their GP due to broad inclusion criteria needed in an effectiveness trial is likely to have reduced the overall power of the study.

To our knowledge, this is the first randomized controlled trial examining the use of a mobile phone self-monitoring program as an intervention tool for young people with depressive symptoms. Self-monitoring was shown to effectively decrease depression via the mechanism of ESA, suggesting that self-monitoring programs that focus on increasing ESA may provide a useful framework for first-step care in depression. The program provided GPs with information about a young person’s daily activities and can be used to detect early signs of mental health problems, such as elevated negative mood, stress and causes of stress, maladaptive coping strategies, isolation from peers, diet, and exercise, as well as other risk and protective factors. The mobile phone self-monitoring program has the advantage of being low cost, quick, and easy to use.

In summary, mobile phones are well suited to first-step interventions, providing an alternative to watchful waiting and allowing young people to provide accurate information to their GPs about their mood and stress [21], as well as shortening the length of time it would take to relay this information to GPs in a usual appointment. Mobile phone self-monitoring programs should be considered as a first-step low-cost intervention with young people who are at risk of mental health problems. Self-monitoring has the advantages of helping young people increase their ESA while gaining more information about their mental health symptoms in order to direct them to the best intervention.

## Multimedia Appendix 1

Emotional self-awareness scale.

## Multimedia Appendix 2

Indirect effect size estimates of the the parallel process latent growth curve model for group on depressive symptoms via the pathway of emotional self-awareness.

## Abbreviations

DASS: Depression Anxiety Stress Scale
ESA: emotional self-awareness
GP: general practitioner
LGCM: latent growth curve model
