# Supplementary material for: Self-monitoring Using Mobile Phones in the Early Stages of Adolescent Depression: Randomized Controlled Trial
Source: J Med Internet Res. 2012 Jun 25;14(3):e67. doi: 10.2196/jmir.1858 (PMC3414872; doi:10.2196/jmir.1858)
Supplement: Supplementary file 1 [file jmir_v14i3e67_app1.pdf]

Emotional Self-Awareness Scale (ESAS)

All items are on a 5-point likert scale ranging from zero to five (0 = Never, 1 = Very Little, 2 = Sometimes, 3 = Often, 4 = A lot). Subscales range from 0 to 20. Total scale ranges from 0 – 132.

Subscales

Recognition: Items 4, 18, 20, 21, 22, 24. Divided by 6. Multiplied by 5.

Identification: Items 1, 3, 8, 17, 29. Divided by 5. Multiplied by 5.

Commuication: Items 6, 12, 13, 15, 27, 30, 3. Divided by 7. Multiplied by 5.

Contextualisation: 5, 7, 10, 11, 14, 16, 19, 28, 32, 33. Divided by 10. Multiplied by 5.

Decision-Making: 2, 8, 23, 25, 26. Divided by 5. Multiplied by 5.

Total ESA score: The sum of all subscales.

Item	Question
1	My moods are hard to describe *(reverse)*
2	I examined my feelings and then decided what to do
3	It’s important to me to understand what my feelings mean
4	It's hard for me to tell what mood I’m in *(reverse)*
5	I analyse my personality to try to understand why I’m upset
6	Expressing emotion is easy
7	I usually know why I feel the way I do
8	I often have trouble deciding what will improve my mood *(reverse)*
9	I know how I feel about most things
10	I don't know why I feel the way I feel *(reverse)*
11	I go away by myself and think about why I feel a certain way
12	I like to write down what I’m feeling and analyze it
13	I can talk about mood to others
14	I don't really think about why I behave as I do *(reverse)*
15	I often 'self-talk' to think about feelings
16	I’m often confused about how I feel about things *(reverse)*
17	I’m often aware of being emotional, but I can’t describe the emotion
18	I frequently take time to reflect on how I feel

19	I often know what caused my mood
20	I’m usually aware of my emotions
21	I like to go someplace alone to think about my feelings
22	I don't often think about my feelings *(reverse)*
23	I often think about ways to make myself feel better
24	I know exactly how I'm feeling
25	Sometimes I can't figure out how to make myself feel better *(reverse)*
26	When feeling bad, I try to deal with my problems and concerns
27	I can verbalise my feelings
28	I usually have clear idea about how my feelings affects my behaviour
29	It’s difficult to make sense of the way I feel about things *(reverse)*
30	I find it easy to write down how I feel
31	It's difficult to communicate what I feel *(reverse)*
32	I often think about the way I feel about things
33	I analyse recent events to try to understand why I’m upset

Note: Reverse scored items are indicted by *(reverse)*
