# Supplementary material for: Self-monitoring Using Mobile Phones in the Early Stages of Adolescent Depression: Randomized Controlled Trial
Source: J Med Internet Res. 2012 Jun 25;14(3):e67. doi: 10.2196/jmir.1858 (PMC3414872; doi:10.2196/jmir.1858)
Supplement: Supplementary file 2 [file jmir_v14i3e67_app2.pdf]

Indirect effect sizes of group on the slope of ESA on the slope of depressive symptoms

The estimates of indirect effect sizes are reported here to allow for comparison with other mediation models, however, these effect sizes are not comparable with commonly used effect sizes.

Indirect effect size	Estimate	95% CI Lower	95% CI Upper
Unstandardised	-.688	-.962	-.487
Partially Standardised	-1.049	-1.35	-.755
Index of mediation	-.517	-.669	-.373
$R2$ recommended by MacKinnon (2008)	.182	.109	.262
$\kappa2$	.540	.426	.640
Effect Size for two groups	-1.189	-.245	-.052

Note: 95% Confidence Intervals reported are bias-corrected and accelerated
